# Safe performance of magnetic resonance of the heart in patients with magnetic resonance conditional pacemaker systems: the safety issue of the ESTIMATE study

**DOI:** 10.1186/1532-429X-16-30

**Published:** 2014-05-06

**Authors:** Christian G Wollmann, Karin Thudt, Bernd Kaiser, Erich Salomonowitz, Harald Mayr, Sebastian Globits

**Affiliations:** 1 Department of Cardiology, Hospital of St. Pölten-Lilienfeld, St. Pölten, Austria; 2 Department of Radiology and Interventional Angiology, Hospital of St. Pölten-Lilienfeld, St. Pölten, Austria; 3 Karl Landsteiner Society, Institute of Cardiovascular Research, St. Pölten, Austria

**Keywords:** Permanent pacemaker, MR conditional, Magnet resonance, Heart MR, Complications

## Abstract

**Background:**

No published data exist about the safety of diagnostic magnetic resonance (MR) of the heart performed in a larger series of patients implanted with MR conditional pacemakers (PM). The purpose of our study is to analyse safety and potential alterations of electrical lead parameters in patients implanted with the EnRhythm/Advisa MRI SureScan PM with 5086MRI leads (Medtronic Inc.) during and after MR of the heart at 1.5 Tesla.

**Methods:**

Patients enrolled in this single center pilot study who underwent non-clinically indicated diagnostic MR of the heart were included in this analysis. Heart MR was performed for analyses of potential changes in right and left ventricular functional parameters under right ventricular pacing at 80 and 110 bpm. Atrial/ventricular sensing, atrial/ventricular pacing capture threshold [PCT], and pacing impedances were assessed immediately before, during, and immediately after MR, as well at 3 and 15 months post MR.

**Results:**

Thirty-six patients (mean age 69 ± 13 years; high degree AV block 18 [50%]) underwent MR of the heart. No MR related adverse events occurred during MR or thereafter. Ventricular sensing differed significantly between the FU immediately after MR (10.3 ± 5.3 mV) and the baseline FU (9.8 ± 5.3 mV; p < 0.05). Despite PCT [V/0.4ms] was not significantly different between the FUs (baseline: 0.84 ± 0.27; in-between MR scans: 0.82 ± 0.27; immediately after MR: 0.84 ± 0.24; 3-month: 0.85 ± 0.23; 15-month: 0.90 ± 0.67; p = ns), 7 patients (19%) showed PCT increases by 100% (max. PCT measured: 1.0 V) at the 3-month FU compared to baseline. RV pacing impedance [Ω/5V] differed significantly at the FU in-between MR scans (516 ± 47), and at the 15-month FU (482 ± 58) compared to baseline (508 ± 75).

**Conclusion:**

The results of our study suggest MR of the heart to be safe in patients with the MR conditional EnRhythm/Advisa system, albeit although noticeable but clinically irrelevant ventricular PCT changes were observed.

## Background

Magnetic resonance (MR) evolved into an important diagnostic tool for diagnosis and therapy control of certain diseases especially affecting soft tissues [1]. Due to safety concerns MR in patients with cardiac implantable electronic devices (CIEDs) is not recommended except in the case of vital indication [2]. Some larger series of MR at 1.5 Tesla (T) in patients implanted with standard pacemakers (PM) demonstrated safety of even repetitive procedures when certain precautions are taken [3-8]. All of the leading pacemaker manufacturers are now offering MR conditional pacemaker systems. Patients implanted with these MR conditional pacemaker systems may undergo MR under certain precautions and conditions [9]. The potentially hazardous MR effects on CIEDs are based on the three components of MR technology, the static magnetic field, the switched gradient magnet fields, and the pulsed radio frequency fields. The effects are dependent on magnet field strength and RF power, and are modulated by patient specific characteristics (e.g. size, anatomy), the patient’s position in the MR bore, as well CIED hardware related characteristics (e.g. lead position, lead design). One major concern in MR in patients with permanent pacemaker and ICDs is lead tip heating resulting in damage of the adjacent cardiac tissue, resulting in PCT increases. Reversion to back up mode is rare but may lead to pacemaker inhibition during MR [4].

The closer the device to the imaging landmark is, the more hazardous on patients health some of the above described MR related effects can become due to deterioration of the implanted systems function and/or behaviour. Nordbeck et al. demonstrated in an in vitro study at 1.5T that electric field magnitudes decrease with reduced RF coupling (and, therefore decreased risk for potential device heating), e.g. by increasing the distance between the area of interest and imaging landmark [10]. Shellock et al. demonstrated in an in vitro model at 1.5T highest temperature increases at the “ventricular” tip electrode when the imaging landmark was within the thorax region of a phantom [11].

To overcome the potentially hazardous effects of MR on pacemaker systems, MR conditional pacemakers have modified material composition (e.g. reduced ferro-magnetic components, Hall instead of Reed switch) and have additional programming features (e.g. one-button deactivation of certain pacemaker functions, asynchronous pacing mode etc.) to ensure safety.

The knowledge about all these possible hazards of MR on CIEDs and lack of sufficiently enough evidence concerning safety of MR scans in the thorax region led to imaging exclusion zones either defined by study protocols for non-MR conditional pacemakers (e.g. MagnaSafe Registry) or by manufacturers for certain MR conditional pacemaker systems (e.g. Medtronic [EnRhythm MRI EMDR01 and Revo MRI RVDR01 with 5086MRI leads], Biotronik [Evia/Entovis family with Safio/Solia leads]) [8,12]. In the meantime further extensive bench testing led to approval outside the US for full body scan with some of the pacemaker systems named above [13,14].

In 2011 2 condensed summaries of the potential hazards of MR on CIEDs, and the initial experience with MR conditional pacemakers have been published [15,16]. Jung et al. concluded: “The availability of MRI-safe technologies reduces the concerns associated with MRI scanning in patients with pacemakers and ICDs”. Shinbane et al. [15] concluded more cautiously that “The design, development, study and implementation of cardiovascular MR conditional devices will continue to require the expertise and collaboration of multiple disciplines and will need to prove safety, effectiveness and cost effectiveness in patient care” [16].

Because of the lack of sufficient data on safety of dedicated diagnostic MR of the heart in patients implanted with MR conditional pacemakers the purpose of our study is to analyse safety and potential alterations of electrical lead parameters in patients implanted with MR conditional EnRhythm/Advisa MRI SureScan pacemakers with atrial and ventricular CapSure Fix 5086MRI lead (Medtronic Inc., Minneapolis, MA, USA) and who underwent dedicated cardiac MR of the heart imaging at 1.5 T.

## Methods

All patients implanted with EnRhythm/Advisa MRI SureScan pacemaker with 5086MRI leads and who underwent MR of the heart within the ESTIMATE Study (*E*rmittlung von ventrikulären Funktion*S*parame*T*ern m*I*ttels *M*agnetreson*A*nz*T*omographi*E* (MRT) während rechtsventrikulärer Stimulation [Magnet resonance imaging guided assessment of ventricular functional parameters during right ventricular pacing]) were included in this analysis. Patients were implanted with permanent pacemakers according to current guidelines [9,17]. Heart MR scans were done for study reasons only. The study was approved by the ethical committee of the government of Lower Austria.

The ESTIMATE study was a prospective single center non-randomized pilot study. Primary endpoint of the study was the measurement of right and left ventricular global functional parameters as well as of regional functional parameters assessed at two different pacing rates (80 bpm and 110 bpm) and a fixed AV delay (110 ms).

Secondary endpoints were assessment of potentially MR related complications during MR (including the occurrence of malignant ventricular arrhythmias), and immediately thereafter as well as in follow-up. Additional secondary endpoints were comparison of atrial and ventricular sensing amplitudes and pacing capture thresholds assessed immediately before MR and thereafter as well as after 3 and 15 months. Within this publication only the results of the secondary study endpoints of the ESTIMATE Study are reported.

### Cardiac MR protocol

All MR scans were performed using an Achieva 1.5 Tesla scanner (release 2.6.3.7, Philips, Netherlands) and a SENSE cardiac coil (5 elements).

All patients were planned to have MR at a pacing rate of 80 and 110 bpm. The isocenter landmark was placed within the thorax. The maximum gradient slew rate (≤200T/m/s [per axis]) and specific absorption rate (SAR; ≤2.0 W/kg) complied with the recommendations given by the pacemaker manufacturer [13,18].

After a reference scan, a balanced fast field echo (B-FFE) with ECG gating in breath hold technique (10-11s, 1 slice per breathhold) for the horizontal long axis (4-chamber view) and short axis of both ventricles was performed. The acquisition parameters were as follows: TR 3.5 ms; TE 1.7 ms.; flip angle 70 degrees; slice thickness 8 mm, gap 1mm; acquired voxel size 2.4 × 1.93 mm, reconstructed voxel size 0.93 × 0.93 mm; 18 heart phases per RR interval. Both ventricles were covered acquiring 12 slices. The total mean scan duration was 2 minutes per FFE sequence. The MR study protocol duration was calculated to last 20 to 30 minutes. SAR levels and scan durations were documented for each of the MR sequences.

Previous to MR all PM were programmed into the SureScan mode according to the recommendations given by the manufacturer [13,18]. Patient surveillance was done by using a combination of a telemetry based ECG and pulse oximetry. All MR scans were supervised by one of the cardiologists of the study group. After the MR scan patients were asked if there were any irregularities (e.g. device vibration).

### EnRhythm/Advisa MRI SureScan pacemaker system

The first permanent pacemaker system ever labelled as MR conditional was the EnRhythm EMDR01 with 5086MRI leads. The pacemaker system was market released in Europe in late 2008. At this time there was an exclusion zone for the placement of the isocenter landmark. The landmark had to be outside vertebrae C1 - Th12. This restriction for the placement of the isocenter landmark was revised in September 2010 when CE mark for full body scan was given. The MR conditional Advisa A3DR01 pacemaker which was market released in Europe in mid 2009 did not have any restrictions concerning the isocenter landmark. Virtually no material differences in components exist between Advisa MRI and EnRhythm MRI except for the battery, which has a small chemical difference (information source: personal communication with Medtronic). Both pacemakers have the same titanium can (volume 12.7 ccm, thickness 8 mm).

The CapsureFix® 5086MRI lead, which is a modified version of CapSureFix® Novus 5076 lead, is a steroid eluting, bipolar, silicone insulated lead with an active fixation mechanism. The lead body diameter is 2.3 mm. Available lengths are 45, 52 and 58 cm, respectively [19]. The major difference to model 5076 is that lead 5086MRI is eligible for the use with magnet resonance, based on modified internal wiring composition to decrease the risk of overheating during an MR scan, and, therefore to reduce potentially dangerous heating at the leads tip [19,20].

### Follow-up

Lead measurements (atrial/ventricular sensing [mV], atrial/ventricular pacing threshold [PCT; Volt/0.4 ms], pacing impedance [Ohms/5V]) and battery voltage (not measured at the follow-up (FU) in-between the MR scans for the different pacing rates) were assessed at 5 in-office follow-ups (immediately before MR, in-between the scans for the 2 different pacing rates, immediately post MR, 3 months post MR, and finally 15 months post MR (3-month FU + 12 months). In addition, clinically relevant parameters (e.g. amount of atrial/ventricular pacing) were retrieved from the pacemakers’ statistics.

### Statistics

Normally distributed continuous data were reported as mean ± standard deviation (SD). One-way analysis of variance was used for comparison of lead measurements between the different follow-ups. Wilcoxon signed ranks test was used to compare pre MR data with the data assessed between the two MR scans (where available) and at each post MR follow-up. A p < 0.05 was considered significant. Analyses were performed using the statistical software package IBM SPSS Statistics 20 (IBM Corporation, Armonk, USA).

## Results

Between Aug 2009 and Aug 2011 thirty-eight patients implanted with the EnRhythm/Advisa MRI SureScan pacemaker system were enrolled in the ESTIMATE study. Two male patients did not undergo MR of the heart within the study (one due to an intrinsic heart rate above 110 bpm, and one due to an elevated arterial blood pressure) and were excluded from further analyses. Therefore, the data of 36 patients are reported. Thirty-one patients underwent MR at both pacing rates, 5 patients at 110 bpm only because of intrinsic heart rates above 80 bpm. Table 1 shows the baseline characteristics of the patient cohort. One third of the patients were female, and the mean age was 69 years. High degree AV block and sick sinus syndrome were the leading pacemaker indications. In one third of the patients the right ventricular lead had been placed in the mid or high septum (Table 1). Six patients were in atrial fibrillation (AF) at the beginning of MR, and one patient developed AF during the MR scan at 110 bpm pacing rate. All pacemakers were programmed to an asynchronous pacing mode as intended by the SureScan mode. All patients with AF at the beginning of MR were programmed to VOO. In 5 patients VOO mode was programmed due to other arrhythmias than AF (e.g. frequent atrial extra beats).

**Table 1:** Patient Demographics

	**N**	**%**
**Total patients**	36	100
**Female**	12	33
**Age (years)**	69 ± 13 [73]	
**Pacemaker indication**		
Higher degree AV block	19	53
Sick sinus syndrome	15	42
Other indication	2	6
**Coronary artery disease**	13	36
**Hypertension**	30	83
**Diabetes**	9	25
**Impaired renal function**	14	39
**Stroke**	5	14
**Pacemaker**		
EnRhythm MRI Sure Scan EMDR01	25	69
Advisa MRI Sure Scan A3DR01	11	31
**PM Diagnostics**		
AP (%; [median])	40 ± 37 [23]	
VP (%; [median])	26 ± 39 [2]	
AF-Burden (%; [median])	17 ± 34 [0]	
**AF before MR**	6	18
**Pacing mode during MR**		
V00	11	31
D00	25	69
**RV lead position**		
apical	23	64
septal	13	36
**Implantation site left pectorally**	35	97
**Time from implantation (months)**	5.3 ± 3.8 [4.5]	

SAR_max_ of the scan sequences was 2.0 W/kg (lowest 1.7; median for all scans 1.9). The cumulative scan duration was 9 min and 54 sec (median 10 min and 35 sec).

### Adverse events

No MR related adverse clinical events occurred. There were no reported malignant arrhythmias, changes in patients’ health status, and no device malfunctions observed during and after MR throughout the observational period. No MR related irregularities (e.g. device vibration) were reported by any of the patients.

### Lead measurements

All patients completed all follow-ups except those patients who had MR at 110 bpm pacing rate only (n = 5) and who therefore didn’t have the follow-up in-between the scans for the different pacing rates. Table 2 displays all measured parameters from all follow-ups.

**Table 2:** Measurements before and after MR of the heart

**Parameter**	**FU immediately before (n = 36)**	**FU in-between (n = 31)**	**FU immediately after (n = 36)**	**3-month FU (n = 36)**	**15-month FU (n = 36)**	**FU immediately before (n = 36)**
Time from MR (months)	-	-	-	2.8 ± 0.7 [3.0]	16.0 ± 3.2 [15.2]	-
Time from implantation (months)	5.3 ± 3.8 [4.5]	-	-	7.9 ± 3.7 [7.6]	21.2 ± 5.7 [20.7]	-
RA Sensing (mV)	2.4 ± 1.3 [2.4]	2.4 ± 1.5 [2.3]	2.3 ± 1.5 [2.0]	2.4 ± 1.3 [2.3]	2.4 ± 1.5 [2.2]	n.s.
RA PCT (V@0,4ms)	0.69 ± 0.22 [0.56]	0.63 ± 0.19 [0.50]†	0.62 ± 0.19 [0.50]	0.69 ± 0.22 [0.50]	0.64 ± 0.22 [0.50]	n.s.
RA Pimp (Ohms)	511 ± 90 [508]	512 ± 100 [504]	503 ± 90 [478]	504 ± 66 [508]	490 ± 72 [484]	n.s.
RV Sensing (mV)	9.8 ± 5.3 [7.5]	10.9 ± 5.0 [10.9]	10.3 ± 5.3 [8.9]†	10.7 ± 4.8 [10.3]	9.9 ± 5.1 [6.8]	n.s.
RV PCT (V@0,4ms)	0.84 ± 0.27 [1.00]	0.82 ± 0.27 [1.00]	0.84 ± 0.24 [1.00]	0.85 ± 0.23 [1.00]	0.90 ± 0.67 [1.00]	n.s.
RV Pimp (Ohms)	508 ± 75 [504]	516 ± 47 [512]†	515 ± 62 [500]	509 ± 84 [494]	482 ± 58 [488]†	n.s.
Battery voltage (V)	3.02 ± 0.03 [3.01]	-	3.01 ± 0.03 [3.00]†	3.0 ± 0.02 [3.00]†	3.00 ± 0.01 [3.00]†	0.024
**Value changes (% [median]; compared with pre MRI FU)**						
RA sensing		5 ± 49 [0]	4 ± 47 [0]	8 ± 48 [0]	8 ± 43 [5]	n.s.
RA PCT		-8 ± 17 [0]	-6 ± 28 [0]	5 ± 40 [0]	-2 ± 38 [0]	n.s.
RA Pimp		1 ± 11 [0]	-1 ± 11 [0]	-0 ± 13 [0]	-3 ± 11 [-6]	n.s.
RV sensing		9 ± 26 [0]	9 ± 20 [4]	17 ± 39 [8]	-3 ± 27 [-6]	0.046
RV PCT		-0 ± 33 [0]	7 ± 38 [0]	12 ± 49 [0]	3 ± 39 [0]	n.s.
RV PImp		3 ± 7 [4]	2 ± 7 [2]	1 ± 14 [0]	-4 ± 10 [-4]	0.015
Battery voltage (V)		-	-0.3 ± 0.3 [-0.2]	-0.4 ± 0.6 [-0.3]	-0.6 ± 0.9 [-0.3]	n.s.

FU…follow-up; PCT…pacing capture threshold; Pimp…pacing impedance.

*Oneway ANOVA.

† p < 0.05 [Wilcoxon signed Ranks Test].

### Sensing

#### *Atrial leads*

No difference for atrial sensing could be shown when comparing all follow-ups (Table 2). Atrial sensing amplitudes measured at the follow-up in-between the MR scans decreased by more than 50% but less than 100% in 3 patients (10%). At the follow-up immediately after MR four patients (11%) had a decrease of the atrial sensing amplitude by more than 50% but less than 100% as compared to baseline. All of these 4 patients were different to the patients who showed a sensing decrease in-between the MR scans. In 1 patient the difference persisted until the 15-month follow-up.

#### *Ventricular leads*

Ventricular sensing differed significantly between the follow-up immediately after MR and baseline (baseline evaluation: 9.8 ± 5.3 mV [7.5]; FU immediately after MR: 10.3 ± 5.3 mV [8.9]; p < 0.05). Compared over all follow-ups ventricular sensing was not different between the follow-ups (Table 2). When comparing the percentages ventricular sensing values changed, a significant difference over all follow-ups could be found showing a proportional increase until the 3-month follow-up, and a proportional decrease afterwards (Table 2).

Two different patients showed RV sensing attenuations by at least 50% (one at the 3- month FU, on at the 15-month FU).

### Pacing capture threshold

#### *Atrial leads*

The atrial PCT assessed in-between the two MR studies was significantly lower than before MR (pre-MR (Volts/0.4ms) [median]: 0.69 ± 0.22 [0.56]; in-between MR: 0.63 ± 0.19 [0.50]; p < 0.05). All other comparisons showed no significant difference between the follow-ups (Table 2). An increase by 100% (max. +0.5V) from the baseline follow-up to the follow-up immediately after MR of the atrial pacing threshold was observed in 1 patient. Three patients (8%), including the one with the early increase, had an atrial PCT increase by 100% (max. +0.5V) at the 3- month follow-up compared to baseline. Only 2 of them maintained the atrial PCT increase until the 15-month follow-up. Of note, the highest atrial pacing threshold amplitude measured at 0.4 ms impulse duration in the course after MR was 1.0 Volt (pre-MR: 1.25 V!).

#### *Ventricular leads*

Pairwise comparison and one-way ANOVA showed no significant differences of the ventricular PCT between the follow-ups (Table 2).

Four patients showed a ventricular PCT increase by 100% (+0.5V [from 0.5V to 1.0 V]) at the follow-up immediately after MR, 2 of these patients showed this increase in-between the scans for the different pacing rates already. A total of 7 patients (19%) had an increase of the ventricular PCT by 100% (+0.5V [highest PCT measured: 1.0V/0.4ms]) at the 3-month follow-up (including all 4 patient who showed this increase immediately after MR), but only 2 of these patients remained having this increase until the end of the observational period. Time from pacemaker implantation to MR of the heart was statistically not different between patients with and without 100% PCT increase (with PCT increase [median]: 4.3 ± 1.9 [5.1] months; w/o PCT increase [median]: 5.3 ± 4.1 [4.1] months; p = ns). SAR_max_ and total scan duration were statistically not different between patients with and without 100% PCT increase. The SAR_max_ was 1.93 ± 0.5 in patients without PCT increase, and 1.93 ± 0.5 in patients with 100% PCT increase, respectively (p = ns). Total scan duration did not differ as well (patients without PCT increase: 10 min 3 sec [median 10 min 35 sec]; patients with PCT increase: 9 min 16 sec [median 10 min 35 sec]; p = ns).

The highest ventricular pacing threshold measured at the 3-month follow-up was 1.25 Volt/0.4 ms (1 patient), and was 1.5 Volt/0.4 ms (1 patient) at the 15-month follow-up, respectively. At the baseline evaluation the highest ventricular PCT measured was 1.5V/0.4 ms (1 patient).

All of the patients except one with the ventricular pacing threshold increase by 100% were implanted with the EnRhythm MRI pacemaker. Two of the patients with the 100% PCT increase at the 3-month follow-up and 1 patient at the 15-month follow-up had a septal ventricular lead position.

### Pacing impedance

Atrial pacing impedance differed significantly between the baseline follow-up and the last follow-up. Ventricular pacing impedance differed significantly between the follow-up in-between the MR scans) and the 15-month follow-up compared to the baseline follow-up (Table 2). Comparing all follow-ups no significant differences could be found. Minimal/maximal values measured were 342 and 896 Ω for atrial leads, and 386 and 832 Ω for ventricular leads, respectively.

### Battery voltage

The battery voltage was significantly different not only between the baseline and the later follow-ups, but also compared between the baseline evaluation and the follow-up that took place immediately after MR (Table 2). Before MR the minimal/maximal voltage measured was 2.96/3.08 Volts, and 2.95/3.06 Volts thereafter, respectively.

### Other parameters

The amount of atrial and ventricular pacing did not differ between the follow-ups at which AP and VP was assessed (baseline FU, 3-month FU, 15-month FU; Table 3). In one patient AF occurred during the MR for the 110 bpm pacing rate. A difference in AF-Burden between the follow-ups could not be found (Table 3).

**Table 3:** Pacemaker statistics

**Parameter**	**Pre MR FU (n = 36) [median]**	**3-month FU (n = 36) [median]**	**15-month FU (n = 36) [median]**	p = *
**Time from MR (months)**	-	2.8 ± 0.7 [3.0]	16.0 ± 3.2 [15.2]	-
**Atrial pacing (%)**	40 ± 37 [23]	42 ± 36 [25]	45 ± 36 [40]	n.s.
**Ventricular pacing (%)**	26 ± 39 [2]	26 ± 38 [3]	30 ± 38 [6]	n.s.
**AF-Burden**	17 ± 34 [0]	15 ± 33 [0]	20 ± 37 [0]	n.s.

FU…follow-up.

*Oneway ANOVA.

### Image quality

**Figure 1:**
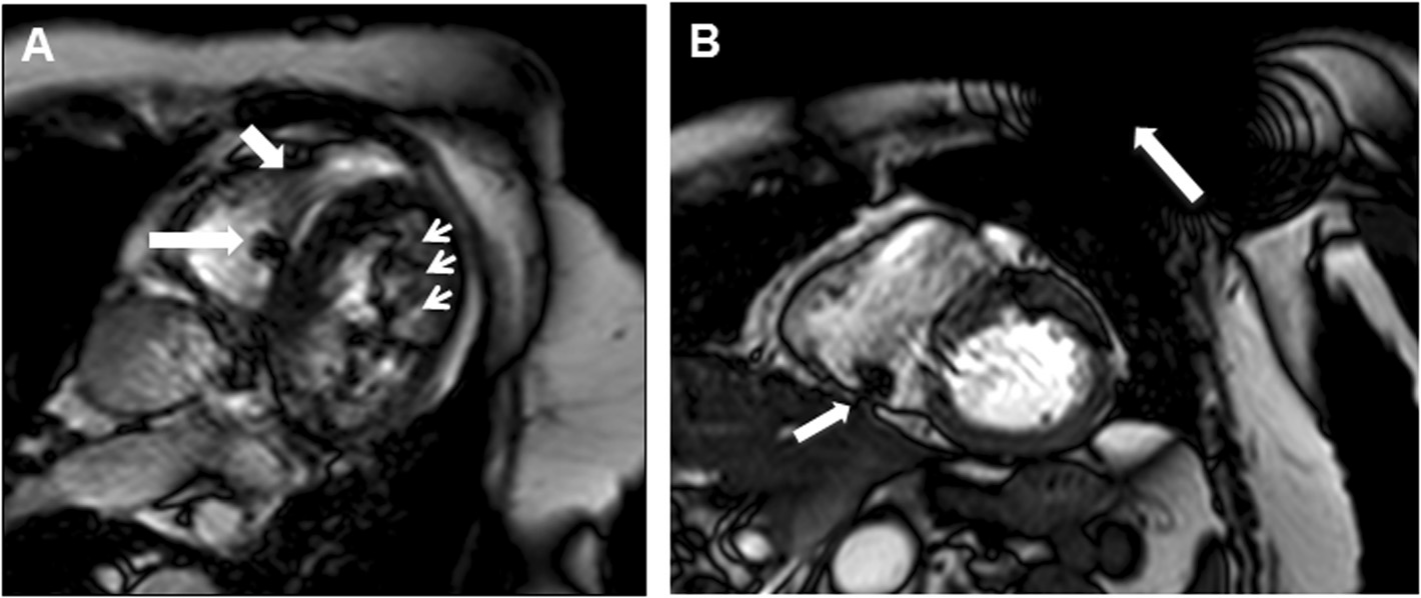
**Device related imaging artifacts. A:** Vertical long axis view showing subtle susceptibility artefacts (short arrow) around the ferromagnetic right ventricular lead (long arrow). In addition, there are prominent off-resonance stripe artifacts in the left ventricle (small arrows) with no impairment of endocardial border detection. **B:** Short axis view with the typical signal void caused by the RV lead (short arrow) in the inferior wall not interfering with the endocardial border. Note the large signal void and circular stripe artifacts caused by the generator (long arrow) with no interference with the heart.

The implantable pulse generator and the leads cause various types of artifacts by magnetic field distortion. Figure 1 shows typical examples. Image quality was graded as 1 = good, 2 = intermediate, 3 = poor. Image quality was good to intermediate in 95% of cases and poor in 5%. However, no cine study was non-diagnostic as a result of hardware related artifacts.

## Discussion

This is the first report on a larger series of patients implanted with MR conditional pacemakers with Medtronic 5086MRI leads who underwent diagnostic MR of the heart. Previously it was demonstrated that MR of the brain and the lumbar spine in patients implanted with the EnRhythm MRI SureScan pacemaker system and the 5086MRI leads was safe [21]. During MR of the heart, and unlike brain MR and lumbar MR the isocenter has to be placed near or within the region of the pacemaker system, which may increase the risk of MR related adverse effects on implanted permanent pacemakers. Rod Gimbel recently reported on chest MR in patients implanted with the Advisa pacemakers with 5086MRI leads [22]. Within this randomized study no MR related adverse events were noted, which can be confirmed by our study. In the study published by Bruce L. Wilkoff et al. there was only one patient in whom an increase of the RV pacing capture threshold was observed at the 1-month post-MR visit [21]. It has been reported that the SAR limit at the lumbar spine scan was exceeded in this patient. In the study reported by Rod Gimbel et al. absolute measured values are not provided. It is reported that no patient had a ventricular PCT increase by more than 0.5 Volts. There were no differences in PCT changes compared to the control group [22].

If the high number of ventricular pacing threshold increases observed in our study is related to the MR procedure remains unclear since there was no control group. Compared to other studies with Medtronic devices the observational period of our study was longer (16 months). Maybe these increases of PCT were not observed within the other studies because of their relatively short follow-up duration.

Two of the seven patients who showed the noticeable ventricular pacing capture threshold increase 3 months after the MR maintained to have this PCT difference until the last follow-up which took place 16 months after the MR on average. In these 2 patients the difference may be due to magnetic resonance. In all the other cases the differences might partly be dependent on the way the thresholds were measured (manual PCT measurement with EnRhythm MRI: 0.5 Volt steps!) or may be purely by chance due to physiological variation. SAR_max_ and total scan duration showed no significant difference compared to patients without 100% PCT increase. The missing association between SAR and changes in lead specific parameters in our study is in line with previously published in vivo data [5,23,24].

Since the 3 month FU took place a median of almost 8 months after device implantation, it is unlikely that the measured value changes assessed within this study are part of the healing process after lead implantation.

In a series of 30 patients implanted with MR conditional single or dual chamber pacemakers of a different manufacturer and who underwent MR of the brain and lumbar spine, no patient showed an atrial and/or ventricular PCT increase by 100% or more [25]. The way the pacemakers used within this study measured pacing thresholds was done in 0.1 Volt steps.

Fact is that only one patient out of the group with 100% PCT increases was implanted with an Advisa pacemaker (manual PCT measurements in 0.25 Volt steps). Besides, the maximal increase of either atrial or ventricular PCT was +0.5V. Septal RV lead position seems not to have a negative influence on PCT after MR. Nevertheless, all of the RV PCT changes observed were in clinically accepted ranges, and – finally - clinically irrelevant. Of note, the maximum measured ventricular pacing capture threshold throughout the study was 1.5 Volts/0.4 ms.

Cardiac MR images were somewhat disturbed by the presence of the IPG and the endocardial leads but offered sufficiently high quality as needed with our analyses (Figure 1). IPG and lead dependent artifacts and their impact on image quality were extensively analysed in a quality evaluation of a large series of cardiac magnet resonance images drawn from a multicenter trial which was conducted to demonstrate the safety and effectiveness of the Advisa MRI pacemaker in an MR environment [22,26]. The recently published results show diagnostic quality in the vast majority of images – as it was within our study [26].

### Limitations

There are some limitations with our study. One limitation is the small number of patients enrolled, and the second limitation is the non-randomized study design. Another limitation is that the enrolled patients had 2 different pacemakers implanted. Therefore, it remains unclear, if the pacing threshold changes observed were MR related or - for example - dependent on the pacemaker specific pacing capture threshold measurement.

## Conclusion

The results of our study suggest the MR conditional EnRhythm/Advisa pacemaker system to be safe when undergoing diagnostic MR of the heart. Except for ventricular PCT the results of our study demonstrate stable lead measurements after MR of the heart performed in patients with specifically for the MR environment designed dual chamber pacemaker systems. The relatively large number of observed ventricular PCT increases may be specific for the manual PCT assessment with EnRhythm MRI pacemakers, but were all clinically irrelevant. No MR related adverse device related or other adverse events occurred within our study.

## Abbreviations

AF: Atrial fibrillation; AP: Atrial pacing; bpm: Beats per minute; CIED: Cardiac implantable electronic device; FU: Follow-up; IPG: Implantable pulse generator; MR: Magnet resonance; ms: Millisecond(s); PCT: Pacing capture threshold; Pimp: Pacing impedance; PM: Pacemaker; RA: Right atrium/atrial; RV: Right ventricle/ventricular; SAR: Specific Absorption Rate; T: Tesla; V: Volt(s); VP: Ventricular pacing.

## Competing interests

Wollmann: Biotronik (lecture fees, travel grants, consulting), Boston Scientific (lecture fees, travel grants, consulting), Medtronic (travel grants, consulting), St. Jude Medical (lecture fees, travel grants, advisory board). Thudt: Biotronik (lecture fees, travel grants), Boston Scientific (lecture fees, travel grants), Medtronic (lecture fees, travel grants). Kaiser: None. Salomonowitz: None. Mayr: Medtronic (lecture fees, travel grants, advisory board). Globits: None.

## Authors’ contributions

CGW: substantial contributions to conception and design; acquisition of data, and analysis and interpretation of data; drafting the manuscript; final approval of the version to be published. KT: acquisition of data, and analysis and interpretation of data; revising it critically for important intellectual content; final approval of the version to be published. BK: substantial contributions to conception and design; acquisition of data, and analysis and interpretation of data; revising it critically for important intellectual content; final approval of the version to be published. ES: substantial contributions to conception and design; acquisition of data, and analysis and interpretation of data; revising manuscript critically for important intellectual content; final approval of the version to be published. HM: substantial contributions to conception and design; revising manuscript critically for important intellectual content; final approval of the version to be published. SG: substantial contributions to conception and design; acquisition of data, and analysis and interpretation of data; revising it critically for important intellectual content; final approval of the version to be published.

## References

[B1] American College of Radiology: Practice Guidelines and Technical Standards [Internet] Available from: http://www.acr.org/Quality-Safety/Standards-Guidelines/Practice-Guidelines-by-Modality/MRI

[B2] Roguin A Schwitter J Vahlhaus C Lombardi M Brugada J Vardas P Auricchio A Priori S Sommer T Magnetic resonance imaging in individuals with cardiovascular implantable electronic devices Europace 2008 10 336 46 10.1093/europace/eun021 18308754

[B3] Sommer T Naehle CP Yang A Zeijlemaker V Hackenbroch M Schmiedel A Meyer C Strach K Skowasch D Vahlhaus C Litt H Schild H Strategy for safe performance of extrathoracic magnetic resonance imaging at 1.5 Tesla in the presence of cardiac pacemakers in non-pacemaker dependent patients. A prospective study with 115 examinations Circulation 2006 114 1285 92 10.1161/CIRCULATIONAHA.105.597013 16966587

[B4] Nazarian S Hansford R Roguin A Goldsher D Zviman MM Lardo AC Caffo BS Frick KD Kraut MA Kamel IR Calkins H Berger RD Bluemke DA Halperin HR A prospective evaluation of a protocol for magnetic resonance imaging of patients with implanted cardiac devices Ann Intern Med 2011 155 415 24 10.7326/0003-4819-155-7-201110040-00004 21969340 PMC4337840

[B5] Martin ET Coman JA Shellock FG Pulling CC Fair R Jenkins K Magnetic resonance imaging and cardiac pacemaker safety at 1.5-Tesla J Am Coll Cardiol 2004 43 1315 24 10.1016/j.jacc.2003.12.016 15063447

[B6] Naehle CP Kreuz J Strach K Schwab JO Pingel S Luechinger R Fimmers R Schild H Thomas D Safety, feasibility, and diagnostic value of cardiac magnetic resonance imaging in patients with cardiac pacemakers and implantable cardioverters/defibrillators at 1.5 T Am Heart J 2011 161 1096 105 10.1016/j.ahj.2011.03.007 21641356

[B7] Naehle CP Zeijlemaker V Thomas D Meyer C Strach K Fimmers R Schild H Sommer T Evaluation of cumulative effects of MR imaging on pacemaker systems at 1.5 Tesla Pacing Clin Electrophysiol 2009 32 1526 35 10.1111/j.1540-8159.2009.02570.x 19793358

[B8] Russo RJ Determining the risks of clinically indicated nonthoracic magnetic resonance imaging at 1.5 T for patients with pacemakers and implantable cardioverter-defibrillators: rationale and design of the MagnaSafe Registry Am Heart J 2013 165 266 72 10.1016/j.ahj.2012.12.004 23453091

[B9] Brignole M Auricchio A Baron-Esquivias G Bordachar P Boriani G Breithardt OA Cleland J Deharo JC Delgado V Elliott PM Gorenek B Israel CW Leclercq C Linde C Mont L Padeletti L Sutton R Vardas PE European Society of Cardiology (ESC) 2013 ESC guidelines on cardiac pacing and cardiac resynchronization therapy: the task force on cardiac pacing and resynchronization therapy of the European Society of Cardiology (ESC). Developed in collaboration with the European Heart Rhythm Association (EHRA). European Society of Cardiology (ESC); European Heart Rhythm Association (EHRA) Europace 2013 15 1070 118 23801827 10.1093/europace/eut206

[B10] Nordbeck P Ritter O Weiss I Warmuth M Gensler D Burkard N Herold V Jakob PM Ertl G Ladd ME Quick HH Bauer WR Impact of imaging landmark on the risk of MRI-related heating near implanted medical devices like cardiac pacemaker leads Magn Reson Med 2011 65 44 50 10.1002/mrm.22592 20806352

[B11] Shellock FG Fischer L Fieno DS Cardiac pacemakers and implantable cardioverter defibrillators: in vitro magnetic resonance imaging evaluation at 1.5-Tesla J Cardiovasc Magn Reson 2007 9 21 31 10.1080/10976640600897237 17178677

[B12] Revo MRI SureScan Pacing System MRI Technical Manual Medtronic, Inc. 2013 [Internet]. Available from: http://manuals.medtronic.com/manuals/main/us/en_US/manual

[B13] EnRhythm MRI SureScan Pacing System Medtronic, Inc. 2011 [Internet]. Technical manual. Available from: http://manuals.medtronic.com/manuals/main/us/en_US/manual

[B14] Biotronik SE&Co KG Press Release August 29,2013 [Internet] Available from: http://www.biotronik.com/wps/wcm/connect/int_web/biotronik/newsroom/press_releases/#jump

[B15] Jung W Zvereva V Hajredini B Jäckle S Initial experience with magnetic resonance imaging-safe pacemakers. A review J Interv Card Electrophysiol 2011 32 213 9 10.1007/s10840-011-9610-0 21993594 PMC3224227

[B16] Shinbane JS Colletti PM Shellock FG Magnetic resonance imaging in patients with cardiac pacemakers: era of “MR Conditional” designs J Cardiovasc Magn Reson 2011 13 63 10.1186/1532-429X-13-63 22032338 PMC3219582

[B17] Epstein AE DiMarco JP Ellenbogen KA Estes NA 3rd Freedman RA Gettes LS Gillinov AM Gregoratos G Hammill SC Hayes DL Hlatky MA Newby LK Page RL Schoenfeld MH Silka MJ Stevenson LW Sweeney MO Smith SC Jr Jacobs AK Adams CD Anderson JL Buller CE Creager MA Ettinger SM Faxon DP Halperin JL Hiratzka LF Hunt SA Krumholz HM Kushner FG al. et American College of Cardiology/American Heart Association Task Force on Practice Guidelines. ACC/AHA/HRS 2008 Guidelines for Device-Based Therapy of Cardiac Rhythm Abnormalities: a report of the American College of Cardiology/American Heart Association Task Force on Practice Guidelines (writing committee to revise the ACC/AHA/NASPE 2002 guideline update for implantation of cardiac pacemakers and antiarrhythmia devices) developed in collaboration with the American Association for Thoracic Surgery and Society of Thoracic Surgeons J Am Coll Cardiol 2008 51 e1 62 10.1016/j.jacc.2008.02.032 18498951

[B18] Advisa DR MRI SureScan Pacing System. MRI procedural information for Advisa DR MRI SureScan.A2DR01 digital dual chamber pacemaker and SureScan leads Medtronic, Inc. 2013 [Internet]. Technical manual. Available from: http://manuals.medtronic.com/manuals/main/us/en_US/manual

[B19] CapSureFix MRI™ SureScan™ 5086MRI Medtronic, Inc. 2012 [Internet]. Technical manual. Available from: http://manuals.medtronic.com/manuals/main/us/en_US/manual

[B20] Medtronic News Release 2008 [Internet] Available from: http://wwwp.medtronic.com/Newsroom/NewsReleaseDetails.do?itemId=1226935170803&lang=de_DE

[B21] Wilkoff BL Bello D Taborsky M Vymazal J Kanal E Heuer H Hecking K Johnson WB Young W Ramza B Akhtar N Kuepper B Hunold P Luechinger R Puererfellner H Duru F Gotte MJ Sutton R Sommer T EnRhythm MRI SureScan Pacing System Study Investigators Magnetic resonance imaging in patients with a pacemaker system designed for the magnetic resonance environment Heart Rhythm 2011 8 65 73 10.1016/j.hrthm.2010.10.002 20933098

[B22] Gimbel JR Bello D Schmitt M Merkely B Schwitter J Hayes DL Sommer T Schloss EJ Chang Y Willey S Kanal E MRI Advisa System Study Investigators Randomized trial of pacemaker and lead system for safe scanning at 1.5 Tesla Heart Rhythm 2013 10 685 91 10.1016/j.hrthm.2013.01.022 23333721

[B23] Mollerus M Albin G Lipinski M Lucca J Magnetic resonance imaging of pacemakers and implantable cardioverter defibrillators without specific absorption rate restrictions Europace 2010 12 947 51 10.1093/europace/euq092 20353963

[B24] Buendía F Cano Ó Sánchez-Gómez JM Igual B Osca J Sancho-Tello MJ Olagüe J Salvador A Cardiac magnetic resonance imaging at 1.5 T in patients with cardiac rhythm devices Europace 2011 13 533 8 10.1093/europace/euq501 21227955

[B25] Wollmann CG Steiner E Vock P Mayr H Monocenter feasibility study of the MRI compatibility of the Evia Pacemaker in combination with Safio S Pacemaker Lead J Cardiovasc Magn Reson 2012 14 67 10.1186/1532-429X-14-67 23009683 PMC3482396

[B26] Schwitter J Kanal E Schmitt M Anselme F Albert T Hayes DL Bello D Tóth A Chang Y van Osch D Sommer T Advisa MRI System Study Investigators Impact of the Advisa MRI pacing system on the diagnostic quality of cardiac MR images and contraction patterns of cardiac muscle during scans: Advisa MRI randomized clinical multicenter study results Heart Rhythm 2013 6 864 72 23434621 10.1016/j.hrthm.2013.02.019

